# The addition of a goal-based motivational interview to treatment as usual to enhance engagement and reduce dropouts in a personality disorder treatment service: results of a feasibility study for a randomized controlled trial

**DOI:** 10.1186/1745-6215-14-50

**Published:** 2013-02-17

**Authors:** Mary McMurran, W Miles Cox, Diane Whitham, Lucy Hedges

**Affiliations:** 1 Institute of Mental Health, University of Nottingham, Triumph Road, Nottingham NG7 2TU, UK; 2 School of Psychology, Bangor University, Brigantia Building, Bangor, Gwynedd, LL57 2AS, UK; 3 Nottingham Clinical Trials Unit, Nottingham Health Science Partners, C Floor, South Block, Queens Medical Centre, Nottingham, NG7 2UH, UK

**Keywords:** Drop out, Personal concerns inventory, Personality disorder, Treatment engagement, Treatment motivation

## Abstract

**Background:**

There are high rates of treatment non-completion for personality disorder and those who do not complete treatment have poorer outcomes. A goal-based motivational interview may increase service users’ readiness to engage with therapy and so enhance treatment retention. We conducted a feasibility study to inform the design of a randomized controlled trial. The aims were to test the feasibility of recruitment, randomization and follow-up, and to conduct a preliminary evaluation of the effectiveness of the motivational interview.

**Methods:**

Patients in an outpatient personality disorder service were randomized to receive the Personal Concerns Inventory plus treatment as usual or treatment as usual only. The main randomized controlled trial feasibility criteria were recruitment of 54% of referrals, and 80% of clients and therapists finding the intervention acceptable. Information was collected on treatment attendance, the clarity of therapy goals and treatment engagement.

**Results:**

The recruitment rate was 29% (76 of 258). Of 12 interviewed at follow-up, eight (67%) were positive about the Personal Concerns Inventory. Pre-intervention interviews were conducted with 61% (23 out of 38) of the Personal Concerns Inventory group and 74% (28 out of 38) of the treatment as usual group. Participants’ therapy goals were blind-rated for clarity on a scale of 0 to 10. The mean score for the Personal Concerns Inventory group was 6.64 (SD = 2.28) and for the treatment as usual group 2.94 (SD = 1.71). Over 12 weeks, the median percentage session attendance was 83.33% for the Personal Concerns Inventory group (N = 17) and 66.67% for the treatment as usual group (N = 24). Of 59 eligible participants at follow-up, the Treatment Engagement Rating scale was completed for 40 (68%). The mean Treatment Engagement Rating scale score for the Personal Concerns Inventory group was 6.64 (SD = 2.28) and for the treatment as usual group 2.94 (SD = 1.71). Of the 76 participants, 63 (83%) completed the Client Service Receipt Inventory at baseline and 34 of 59 (58%) at follow-up.

**Conclusion:**

Shortfalls in recruitment and follow-up data collection were explained by major changes to the service. However, evidence of a substantial positive impact of the Personal Concerns Inventory on treatment attendance, clarity of therapy goals and treatment engagement, make a full-scale evaluation worth pursuing. Further preparatory work is required for a multisite trial.

**Trial registration:**

ClinicalTrials.Gov.UK Identifier - NCT01132976

## Background

Good outcomes in psychotherapies depend upon service users’ engagement in those therapies and their adherence to the treatment program [1,2]. Non-completion of treatment is an important matter for mental health services generally, and personality disorder (PD) treatment services in particular. A systematic review of non-completion of psychosocial treatments for PD identified that, on average, 37% of those starting therapies did not complete their treatment [3]. This review also identified studies that showed non-completion of treatment to be associated with poorer clinical outcomes. Compared with treatment completers, those who did not complete treatment were shown to be hospitalized more frequently and to spend more days in hospital [4,5]. Poor attendance also compromises service efficiency and cost-effectiveness through poor use of resources and consequent increased costs associated with treatments [1]. To reduce treatment non-completion, efforts need to be made to enhance service users’ treatment motivation and increase their readiness to engage with PD therapy.

While attending to service user engagement is a core and continuing task in therapy [6], pre-therapy preparation also has a role to play. In a recent systematic review of strategies for reducing drop-out rates in psychotherapy, 15 empirical studies were identified, of which 12 were pre-therapy preparation (for example, role induction, experiential pre-training), and half of these studies had positive outcomes on retention in treatment [7]. The authors of the review commented on a need to identify strategies that are effective with specific groups of patients and mentioned that ‘patients with severe personality disorder are notoriously difficult to keep engaged in treatment … Identifying effective strategies for keeping these patients in therapy would have a major clinical impact’ [7].

One promising approach that may assist the therapist to motivate people to engage in therapy and effect positive change is based upon goal theory. An individual’s personal goals are what give purpose, structure and meaning to a person’s life [8], and well-being is experienced when there is commitment to goal attainment, goals are achievable, and goals meet the individual’s explicit and implicit needs [9,10]. These needs range from basic physiological needs (for example, for oxygen, food, water), through needs for safety and belonging, to higher-order needs for esteem and self-actualization. Clarification of a person’s valued goals, identification of the obstacles to goal attainment, and drawing attention to where therapy might help overcome those obstacles may provide individuals with the motivation needed to enter and engage with therapies.

One specific theory of motivation in which goal striving plays a central role is the Theory of Current Concerns [11]. Within this framework, each goal pursuit corresponds to an internal state called a ‘current concern’. In an interview called the Personal Concerns Inventory (PCI) [12], goals are identified and rated on scales of value, attainability, control and commitment. The rating scales provide information that enables the calculation of indices representing a person’s motivational structure, and empirical investigations have revealed adaptive and maladaptive motivational profiles [13,14]. Adaptive motivation is characterized by high perceived likelihood of goal attainment, expected happiness when goals are attained and commitment to goal striving. It is predictive of readiness to change and an ability to reduce problematic behavior. Although the PCI is an assessment of goals and motivational structure, the experience of clarifying one’s goals can be beneficial in itself, and there is some evidence that engaging in the interview may motivate people to enter treatment [15]. We aimed to capitalize upon this effect by using the PCI procedure as the basis of a motivational interview intended to enhance treatment motivation and engagement and so improve retention of service users in treatment. This effect needs to be evaluated in a randomized controlled trial (RCT). In preparation for this, we conducted a pilot study in a PD treatment service to examine the feasibility of recruitment, randomization and follow-up. Additionally, we aimed to conduct a preliminary evaluation of the effectiveness of the motivational interview. The protocol was published in a previous paper [16]. Specifically, we aimed to measure the recruitment rate to the PCI interview plus treatment as usual (TAU) and TAU only, assess the completeness of follow-up data collection, assess the acceptability of the intervention to clients and therapists, and examine the degree of difference between groups on goal clarity, treatment motivation and treatment retention.

## Method

### Design

The study used a single center, randomized, parallel-group design at the outset, with the addition of a second site at a later stage to overcome recruitment problems.

### Ethics

Approval for the research was given by the Leicestershire, Northamptonshire and Rutland Research Ethics Committee 1 (Ref: 09/H0406/76) and Nottinghamshire Healthcare NHS Trust’s Research Management and Governance Section (Ref: CSP/18/05/10 CSP ID 19434).

### Participants and recruitment

The plan was to conduct the research in Nottinghamshire Healthcare NHS Trust’s Personality Disorder Network, an outpatient service for people with PD. In preparing for this research, we identified that this service offered psychological treatment to 354 people between 2005 and 2008. Of these, 31% dropped out of treatment prematurely. This treatment consisted of giving information and advice, followed by either a 16-week psychological intervention based upon psychoeducation, social problem solving and emotion regulation, or a long-term, day therapeutic community. We planned to recruit from referrals accepted to the psychological intervention. The number of people assessed for psychological treatment by the service was 118 per year; we aimed to recruit 100 participants over 18 months. This enabled a fair test of the feasibility criteria and, if recruitment was good, potentially permitted a reliable calculation of the effect size of the intervention for computing the later sample size.

Those individuals who were accepted for psychological treatment were informed about the project by clinical staff and asked if they would be willing to speak with the researcher to receive further information, when they were fully informed about the research and given an information sheet. For those agreeing to participate, consent was taken by the individual’s clinician at the next appointment. After the clinical assessment period concluded, participants were randomized to receive the PCI interview plus TAU or TAU only. The PCI interview was to be completed by the service’s therapists, all of whom were trained in its delivery. These therapists also delivered TAU to all participants.

### Randomization and blinding

Randomization was created using Stata SEv9 (StataCorp, College Station, TX, USA) statistical software with a 1:1 allocation using random permuted blocks of varying size prepared by the Nottingham Clinical Trials Unit statistician and held on a secure server. Participants were randomly assigned to one of two treatments groups by means of a web-based randomization system accessed by an assistant psychologist after clinicians had obtained consent. Participants and therapists were aware of the intervention allocation. The protocol stated that the research assistant responsible for collecting the outcome measures was kept blinded to the allocation until trial-related assessments were complete.

### Interventions

The comparison groups were PCI plus TAU and TAU only. Participants recruited to the PCI group received an interview of approximately 1.5 hours duration in addition to TAU. The PCI procedure asks participants to identify their goals in 11 life areas (for example, relationships, work or education, home, health), and then prioritize five goals. These five goals were then rated on scales from 0 to 10 assessing five aspects of goal attainment: likelihood of attainment, control over attainment, knowing how to attain it, happiness upon attainment, and commitment to attaining it. Participants were then asked to identify obstacles to goal attainment and consider the possibility that therapy could help them overcome these obstacles. This was intended to enhance participants’ motivation to engage in therapy. Initially, the usual treatment consisted of a maximum of four individual weekly sessions of psychoeducation, based on personality assessment and information exchange [14], after which there was a weekly problem-solving therapy group lasting 12 weeks [15]. However, changes to the service structure during the project, and later recruitment of an additional site, meant that TAU varied from this original plan.

### Assessments

1. **The Personality Diagnostic Questionnaire-4** (PDQ-4) [17] was selected to describe the PD profile of the sample because it was part of the Nottingham PD service’s routine assessment. The PDQ-4 is a 100-item, self-administered, true or false questionnaire that yields personality diagnoses consistent with the Diagnostic and Statistical Manual, Fourth Edition diagnostic criteria for the Axis II disorders.

2. **A pre-intervention interview** was conducted with participants in both groups within the two weeks prior to the start of the therapy group. The purpose was to briefly assess the goals that they expected therapy to help them achieve in two questions: ‘What in general do you expect to get out of therapy?’ and ‘What specific goals do you want to achieve in therapy?’

3. **Attendance records** were kept for group therapy sessions.

4. **The Treatment Engagement Rating scale** (TER) [18] was completed by therapists for each participant at the end of group therapy. This scale contains 22 items in nine scales, each of which is a mean score of the constituent items rated on a scale of 1 to 5, with higher scores indicating greater engagement. The scales are: participation, making sacrifices for treatment, openness, efforts to change problem behavior, goal directedness, efforts to change socio-economic situation, constructive use of sessions, dealing with the content of therapy between sessions, and a global assessment of engagement. The TER total score is the mean of the nine scale scores and ranges from 1 to 5.

5. **The Client Service Receipt Inventory** (CSRI) [19] was used to capture recent use of health and social care. The CSRI was administered by clinicians as routine data collection at baseline and again at the end of treatment.

6. **A post-intervention interview** was conducted with participants in the PCI group, both treatment completers and non-completers, asking for their views on the acceptability and usefulness of the interview. Five questions asked about their general opinions of the PCI interview, benefits from participating, disadvantages from or dislikes about participating, effects on treatment engagement, and a rating of usefulness on a scale of 0 to 10 (higher scores indicate more useful).

7. **A therapist interview** was planned to assess therapists’ opinions of the PCI.

### Planned analyses

The criteria for feasibility of an RCT were: a recruitment rate to the project of 54% of all referrals, based upon the recruitment rate of 54% in another treatment trial of community PD adults in the same locality [20]; 80% of clients finding the intervention acceptable in terms of its practicability and usefulness; and 80% of therapists finding the intervention helpful.

In a full-scale RCT, the primary outcome measure will be completion of treatment, that is, completion of ≥75% of sessions offered. Therefore, the plan was to collect information on attendance at therapy sessions and completion of treatment. The feasibility of examining the processes of engagement was tested by assessing the clarity and specificity of therapy goals pre-treatment, and engagement during treatment, which was rated at the final follow-up by therapists using the TER. The use of the goal-rating scale and the TER will be considered feasible if 80% of participants have these completed for them. The costs associated with the intervention were identified, and the feasibility of calculating the cost-benefits of the intervention was tested by data capture using the CSRI. The CSRI will be considered feasible if 80% of participants have completed the CSRI at baseline and 80% have completed the CSRI at follow-up. The views of clients and therapists on the intervention, collected using semi-structured interviews, were analyzed using thematic analysis [21].

### Changes to the protocol

Over the duration of the project, there were several major unanticipated and uncontrollable events in the service where we were recruiting participants. These led to changes in the protocol, the inclusion of an additional site for participant recruitment, and an extension of the recruitment period. The Ethics Committee approved all changes to the protocol and materials. The specific challenges and responses were as follows.

Significant changes were made to the organization of the service, including replacement of the four-session individual assessment by a 12-session assessment and preparation group, and additional therapies being added to the treatments offered. Changes were made to the protocol regards the timing of the approach to potential participants about the research and changing the measure of sessions attended to include whichever group the service user joined. A three-month planned break of recruitment was agreed while the service implemented its changes and ethical approval for protocol changes was acquired.

During the study period, there were staff departures and long-term leave (for example, maternity leave). The lead psychologist and research co-investigator left the service seven months into the project. A temporary freeze on staff recruitment left posts unfilled, causing pressure on the time of those staff remaining. To cope with this, a temporary freeze on referrals to the service was put in place and there was a partial withdrawal of services, thus reducing the pool of potential participants. Because of the time pressures, the service withdrew clinical staff from conducting the PCI interview. Our response was to involve the local Mental Health Research Network (MHRN) to help with recruitment and the participant follow-up interview, and to deploy the research assistant to conduct the PCI interviews. Because recruitment to the project was below target, we asked for and were granted a five-month, within-cost extension to the project. We also recruited a second site to the project - the Leeds Personality Disorder Network. Because the main objective was to test recruitment and randomization, we continued recruitment up to four weeks before the project ended. This meant that data collection concluded before all data were collected on some participants.

In summary, the major changes to the protocol were as follows:

1. Clinicians could not administer the PCI, as planned. The research assistant took over administering the PCI. Clinicians were not blind to whether the PCI was conducted or not.

2. The research assistant could not conduct the pre-intervention interview that required her to be blind to participants’ group assignment, and so MHRN staff conducted this interview.

3. The reorganized service included groups that focused on assessment, and therapy groups that were of longer duration than the project. Therefore, it was not possible to assess treatment completion. Instead, we assessed sessions attended over a 12-week period after the PCI had been administered or an equivalent period of time.

4. Because the research assistant conducted the PCI, she could not conduct the post-intervention interview, which asked participants their experiences of the PCI. Thus, MHRN staff conducted this interview.

5. Because the therapists did not conduct the PCI, their views could not be collected.

6. The Leeds site did not complete the PDQ-4 as routine practice. No specific PD information is available from this site.

7. Recruitment was extended from 18 months to 23 months.

## Results

### Participants

Participants are described in Table 1.

**Table 1:** Characteristics of participants

	**Variable**	**Group**	
		**PCI (N = 38)**	**TAU (N = 38)**
Sex	Male	11 (28.94%)	16 (42.10%)
	Female	27 (71.05%	22 (57.89%)
Mean age (SD)		34.53 (9.71)	35.79 (10.52)
Ethnicity	White	34 (89.47%)	34 (89.47%)
	Other	2 (5.26%)	1 (2.63%)
	Not recorded	2 (5.26%)	3 (7.89%)
Employment	Employed	5 (13.16%)	4 (10.53%)
	Unemployed	22 (57.89%)	27 (71.05%)
	Retired	1 (2.63%)	1 (2.63%)
	Not recorded	10 (26.32%)	6 (15.79%)
Diagnosis*	Paranoid	19 (50.00%)	16 (42.10%)
	Schizoid	12 (31.58%)	9 (23.68%)
	Schizotypal	16 (42.10%)	9 (23.68%)
	Histrionic	7 (18.42%)	0 (0%)
	Narcissistic	4 (10.53%)	3 (7.89%)
	Borderline	20 (52.63%)	18 (42.37%)
	Antisocial	14 (36.84%)	11 (28.95%)
	Avoidant	20 (52.63%)	19 (50.00%)
	Dependent	14 (36.84%)	10 (26.31%)
	Obsessive-compulsive	14 (36.84%)	12 (31.58%)
	Negativistic	14 (36.84%)	16 (42.10%)
	Depressive	20 (52.63%)	13 (34.21%)
Referrer	Crisis resolution team	2 (5.26%)	4 (10.53%)
	Community mental health team	27 (71.05%)	20 (52.63%)
	Discharge from inpatient care	0 (0%)	0 (0%)
	Assertive outreach team	0 (0%)	0 (0%)
	Psychology service	1 (2.63%)	0 (0%)
	General practitioner	3 (7.89%)	4 (10.53%)
	Not recorded	5 (13.16%)	10 (26.32%)

PCI: Personal Concerns Inventory; SD: standard deviation; TAU: treatment as usual.

* Note: A person may have more than one diagnosis.

### Recruitment

**Figure 1:**
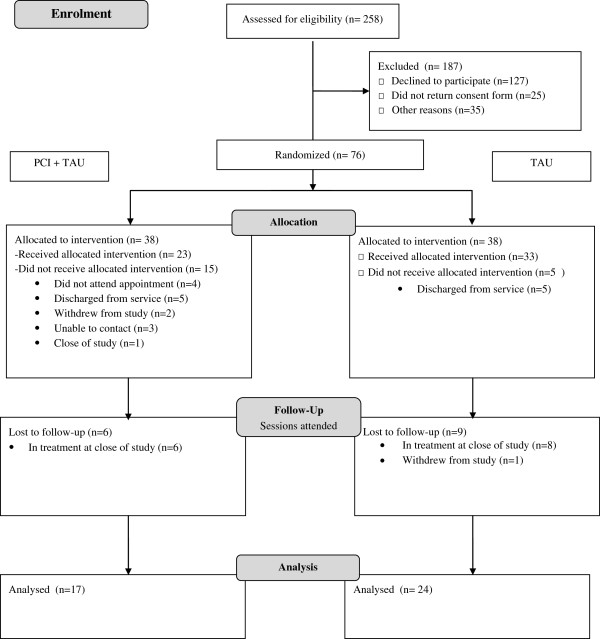
CONSORT flow diagram.

The criterion for feasibility of a full-scale RCT was that recruitment to the project would be 54% of all referrals. The actual recruitment rate was 29% (76 of 258). Of the 76, 38 were randomized to receive the PCI interview and 38 to TAU. The CONSORT diagram is presented in Figure 1.

### Acceptability to service users

The criterion for the feasibility of a full-scale RCT was that 80% of clients would find the intervention acceptable in terms of its practicability and usefulness. Of the 38 randomized to the PCI group, interviews were collected from 16 (42%). However, nine people were ineligible for this interview because they were still in the treatment phase. Of the remainder, five were unavailable because they had been discharged from the service, five did not attend the interview, two withdrew from the study, and one could not be contacted. Interviews for a further three participants were excluded because they did not focus on their experiences of the PCI; an inexperienced interviewer asked them to focus on treatment in general. Thus, 13 interviews were examined.

Participants were asked, ‘Do you think the session(s) did anything to help you become more strongly engaged?’ Of the 13 respondents, six (46%) answered *yes*, four (31%) said *no*, two (15%) said *possibly*, and one (8%) *could not remember*. Overall, of the 12 who could remember the interview, eight (67%) were positive. This falls somewhat short of the criterion of 80%. Participants were also asked to rate the usefulness of the interview on a scale of 0 (not at all useful) to 10 (very useful indeed). Ratings were collected from 11 of the 13 participants (85%). The mean was 7.82 (SD = 1.94), and the median and mode were both 8.

### Session attendance

Over 12 weeks, the PCI group (N = 17) was offered a mean 10.65 (SD = 2.76) sessions, of which they attended a mean of 8.18 (SD = 3.56). The TAU group (N = 24) was offered a mean 9.71 (SD = 3.39) sessions and attended a mean 6.54 (SD = 4.75). The median percentage of attendance by sessions offered was 83.33 (interquartile range: 0.50, 91.67) for the PCI group and 66.67 (interquartile range: 27.50, 91.67) for the TAU group. The percentage value was first transformed using the arcsine of the square root of the percentage value, then Cohen’s *d* was calculated as usual using the transformed value. The effect size (Cohen’s *d*) of the difference in session attendance is 0.44 (95%CI: 0.30, 0.57), which is considered a medium effect.

### Processes of engagement

The feasibility of examining the processes of engagement was tested by assessing the specificity and clarity of goals pre-treatment, and engagement post-treatment.

#### Pre-intervention goals

Pre-intervention interviews were conducted with 61% (23 of 38) of the PCI group; seven were discharged from the service before the interview could take place, five did not attend the interview, and three were not interviewed before the close of the study. Of the TAU group, 74% (28 of 38) were interviewed; four were discharged from the service before the interview could take place, and six did not attend the interview. Again, this falls short of the target of 80%. The goals generated by participants were blind-rated by the senior clinicians (MM and WMC) for clarity and specificity on a scale of 0 (not at all clear or specific) to 10 (clear and specific). This information indicates whether the PCI works better than TAU to clarify clients' thinking about their therapy goals. The mean score for the PCI group was 6.64 (SD = 2.28) and that for the TAU group was 2.94 (SD = 1.71). The effect size (Cohen’s *d*) of this difference is 1.86 (95%CI: 1.20, 2.52), which is considered a large effect.

#### Treatment Engagement Rating scale

Of the total 76 participants, 40 completed the TER (53%). These were 19 of 36 in the PCI group (50%) and 21 of 38 in the TAU group (55%). However, at follow-up, 17 of the 76 (22%) were still in the treatment phase and were not eligible for TER. Therefore, of eligible participants at follow-up, 40 of 59 (68%) completed the TER - 19 of 29 (66%) of whom were in the PCI group and 21 of 30 (70%) in the TAU group. This again falls short of the target of 80%. However, the mean TER score for the PCI group was 3.35 (SD = 0.30) and that for the TAU group was 2.83 (SD = 0.34). The effect size (Cohen’s *d*) of this difference is 1.62 (95%CI: 0.90, 2.33), which is considered a large effect.

### Costs of treatment

Of the total 76 participants, 63 (83%) completed the CSRI at baseline. These were 32 of 38 in the PCI group (84%) and 31 of 38 in TAU (82%). At follow-up, 34 of 76 (45%) completed the CSRI - 18 of 38 (47%) in the PCI group and 16 of 38 (42%) in the TAU group. However, at follow-up, 17 of the 76 (22%) were still in the treatment phase and hence were not eligible for CSRI follow-up. Therefore, of eligible participants at follow-up, 34 of 59 (58%) completed the CSRI; 18 of 29 (62%) were in the PCI group, and 16 of 30 (53%) were in the TAU group. Thus, CSRI data collection met the 80% target at baseline but not at follow-up. The PCI took an average 1.5 hours to complete. According to NHS Reference Costs [22] a face-to-face assessment by a member of a specialist outpatient team costs £145 (range £103 to £158).

### Views of service users

The views of 13 service users, collected using semi-structured interviews, were analyzed using thematic analysis [21]. Four themes were identified.

#### Specific benefits

Participants reported that the PCI interview ‘got them thinking’. Specifically, it helped clarify goals.

*It was quite informative actually because I hadn’t thought about setting myself any goals before it.* [P014]

*It really got me thinking about control and knowledge especially. Overall it was really good to think about my goals before I started.* [P037]

*A lot more in depth than I thought it would be but it sorted out the wheat from the chaff and got down to a reason.* [P044]

This stimulation of thinking produced a number of beneficial effects. In relation to the purpose of this research, it was reassuring to hear the frequently expressed view was that the process helped people to focus on the issues to work on in therapy and get more out of therapy.

*Helped focus on the issues that I needed to work on.* [P005]

*It made me understand what I wanted from the group.* [P019]

*With [the interviewer’s] help I was able to identify and achieve targets that were appropriate. The goal-setting interview led to getting a lot out of the therapy sessions.* [P024]

*It got me thinking before I attended the group so I had like a head start I guess. I had pre-thoughts before the group.* [P021]

*It made me more engaged because it gave me goals to work towards. It made me think about things to focus on to improve my life.* [P052]

*Identifying the goals made the reason for going into DBT [dialectical behavior therapy] very clear.* [P059]

Identifying important goals also engendered positive feelings about the future.

*Felt uplifted by the interview about what my future could be. Just the process was good and I still have those goals in my mind to achieve in time.* [P008]

*It reinforced for me my reasons to get well.* [P052]

This process was not always comfortable:

*Double-edged sword – a shocker that it brought things up but it put things into focus at the same time.* [P005]

*Some of the questions were quite hard to think about*. [P021]

*It was quite hard going and very detailed and some of it hard, but that’s not to say not worthwhile.* [P059]

#### General benefits

The interview also had a more general beneficial effect of preparing people for therapy. It was seen as helpful for familiarizing individuals with the location where therapy would take place.

*Helped break the ice of the environment I would be going into for treatment sessions.* [P005]

*Just coming into the center beforehand helped as familiar with the center then and met members of staff.* [P037]

The individual interview was also appreciated as raising confidence for participation in subsequent group work.

*It was nice to have that one-to-one time. It was a chance to get everything out of my mind before starting. It felt like a safe place as not in a group situation*. *It gave me more confidence to come into the group and talk about things I’ve never discussed before.* [P037]

*I think it made me realize how important what I was going to be doing was because I realized in a way it made me prepared to be more open from the beginning.* [P052]

*Helps get the eye in a bit more - makes you feel confident as to why you are there.* [P059]

There was relief in speaking about past problems with attending treatment.

"I gave [the interviewer] a lot of information of what happened to me and why I hadn’t attended sessions before … so it was a bit of a release really. [P006]"

Some participants offered little precision in their views, but expressed a generally positive feeling about the interview.

*Yes it was ok. I understand why they did it and it was a good idea but… I don’t really know what else to say.* [P019]

*It was good, no complaints. I can't remember. I came away thinking it was good. I would remember if I didn't.* [P048]

#### Interviewer characteristics

Many participants expressed appreciation of the interviewer’s interpersonal style and skills.

*[The interviewer] was really positive. She wasn’t forceful but managed to get over what she wanted me to do. She was very supportive to me and had a lovely tone*. *[She] talked to me properly … not like a child like most people do … I wish other people in mental health field were more like her then we might all be better.* [P008]

*[The interviewer] was fantastic and although I was anxious [the interviewer] was very helpful and understanding, making me feel at ease and positive because [the interviewer] understood my anxiety problems. I felt not rushed and that if necessary I could ask for further explanation.* [P024]

*Personality and demeanor of person that does interview is very important. [The interviewer] was the perfect person, very soft gentle, intuitive - very sensitive to my needs. The interviewer is very important.* [P059]

#### Other problems or concerns

In some cases, other problems or other concerns were more pressing and the PCI was either seen negatively or not remembered.

*I just found it very intrusive. I wasn’t fussed about it. Summat [something] was going on in my personal life, things went off and I went into myself and I just couldn’t cope.* [P053]*Can't remember. I'm sure it did but I've got a lousy memory. I often get depressed and hit the drink and it just clears the memory.* [P048]

*I did find it very useful but my health got in the way of me going so I had to drop out.* [P019]

Additionally, two participants made suggestions for improving the process.

*It would have been good to have a copy of it to take away to see what I have achieved.* [P037]

*It was very useful but partway through it would have been helpful to have a second one-on-one interview to see if my goals had changed at all.* [P052]

## Discussion

Overall, the study fell short of its targets. At 29%, recruitment was below the target of 54% of referrals to the service. Even after an extension of the recruitment period from 18 months to 23 months, only 76 people were recruited - 24% short of the target of 100 recruits. At follow-up, 58% of TER scores were obtained (target 80%) and 58% of CSRI (target 80%). Reasons for the shortfall were largely unanticipated and uncontrollable changes to the service in which recruitment took place. A major influence on recruitment at any research site is the support given by managers and staff. Research is viewed as a non-essential activity, and essential issues need to be satisfactory before research can be given adequate support [23]. Over the 32 months of this project, major and unexpected challenges were experienced: changes to the treatments provided; a freeze on staff recruitment; withdrawal of services in some localities; freeze on receipt of referrals to the service; long waiting times for service users to get into group treatment; withdrawal of clinicians’ time to conduct the PCI; and departure of the site co-investigator. The research was conducted in a turbulent service and this no doubt had an impact on recruitment and retention.

Balanced against recruitment problems is evidence that most service users who were in a position to report their experiences found the PCI acceptable (67%; target 80%) and they rated the PCI’s usefulness as 8 out of 10. Opinions revealed that the PCI benefited engagement both through specific processes, helping people to clarify important personal goals that therapy could help them attain, and general processes, such as familiarizing people with the venue and personnel providing therapy services and building confidence for starting therapy. The characteristics of the interviewer were highly relevant to perceived beneficial effects, with interviewees expressing appreciation for a respectful and sensitive style. Those who did not complete the interview may have had less favorable views.

Perhaps the most persuasive argument for suggesting the need for a full RCT in the face of recruitment difficulties is the evidence of a significant positive impact of the PCI on treatment attendance (Cohen’s *d* = 0.44), clarity of therapy goals (Cohen’s *d* = 1.86), and treatment engagement (Cohen’s *d* = 1.62), although treatment engagement was rated by unblinded therapists, which may account for this effect. At a cost of only £145 per session, a full-scale evaluation of the PCI as pre-therapy preparation seems well worth pursuing. The substantial effect sizes also indicate that the sample size required to test the effectiveness of the PCI interview will not be impractically large. The question is whether we have learned sufficient about the implementation of procedures to be confident of the viability of a full-scale trial.

The first lesson relates to the risk inherent in relying on a single site. A multisite trial would offer a degree of protection against unanticipated and uncontrollable organizational changes that can thwart even the best designed of trials. However, the design of a multisite trial would need to take into account variations in practices between sites.

Furthermore, site selection is of critical importance. Typically, sites are selected on objective criteria, such as the number of eligible patients. Although these are clearly important, other, more subjective criteria should also be taken into consideration, such as clinicians’ enthusiasm for trial participation, support from clinical directors and service managers, and the leadership qualities of the site co-investigators [24]. Gauging the stability of the service also requires attention.

Another important lesson relates to the timing of the initial approach to potential participants. We made this during the assessment phase, when service users were giving and receiving large amounts of information. Overload at this time led many people to turn down the invitation to meet with the researcher on the grounds that they did not want to volunteer for the extra assessments and input associated with the research. The initial contact to inform service users about the research should be made before or after the most intensive assessment period. Most of those who agreed to meet with the researcher to find out more about the project consented to participate.

We also learned lessons about the delivery of the PCI interview. The timing of delivery of the PCI was important, in that adverse conditions such as current physical and mental health problems meant that people were not receptive to the PCI interview. The PCI interview does ask about health and medical matters that may be of current concern to interviewees, but it may be that certain problems prevent people engaging from the start so that they do not get to these questions. After describing the purpose of the PCI interview, it might be useful to ask a preliminary question about any issues that may prevent the interviewee from engaging in the procedure. The addition of written materials to accompany the interview is likely to be of benefit, and a booklet summarizing the procedure in general and recording the individual’s own responses should be produced.

In an effectiveness trial, it will be important to rule out alternative explanations for any observed effect. Clearly, care needs to be taken that blinded researchers collect follow-up data. Blinding the therapists who provide the usual treatment to whether or not the participant has undertaken the PCI interview would rule out biases in staff ratings of participant engagement in therapy. Reliance on dedicated PCI interviewers may not reflect how services would operate in actual clinical practice, but this might be a useful procedure for controlling the quality of the PCI interviews.

Given these uncertainties, further preparatory work is required to estimate the likely rates of recruitment and retention of participants in a multisite trial, and to properly test the acceptability and viability of procedures such as the delivery of the PCI by clinicians and the blinding of researchers. This is in accordance with the Medical Research Council guidance on the evaluation of complex interventions. The Medical Research Council recommends thorough piloting and feasibility work to be confident that the intervention can be delivered as intended, safe assumptions about effect sizes and variability can be made, and rates of recruitment and retention can be gauged prior to designing a main evaluation study [25]. Full-scale evaluations are expensive and preparatory work through a series of studies may be required to progressively refine the design before embarking on a full-scale evaluation. Conducting a pilot study of the PCI evaluation is the next step.

## Conclusions

This study indicates that the intervention has substantial potential value for helping people with PD remain in treatment, at least in the short term. At £145, the cost of the single session interview may be a good investment against the potential adverse effects of treatment non-completion. The purpose of this study was to examine the feasibility of a full-scale trial. Important lessons have been learned for increasing the chances of achieving recruitment and retention targets. Nonetheless, additional piloting is needed to prepare adequately for a multisite trial. On an optimistic note, a number of PD services have expressed an interest in participating in future evaluation studies.

## Abbreviations

CSRI: Client receipt service inventory; MHRN: Mental health research network; PCI: Personal concerns inventory; PD: Personality disorder; PDQ-4: Personality diagnostic questionnaire-4; RCT: Randomized controlled trial; TAU: Treatment as usual; TER: Treatment engagement rating scale.

## Competing interests

The authors declare that they have no competing interests.

## Authors’ contributions

MM, WMC and DW contributed to the design of the study. All authors contributed to the creation of the manual of procedures and the study protocol. LH conducted the research. MM drafted the manuscript. All authors provided a critical review and final approval of the manuscript.
